# Perceived Cognitive Demands of Emotion Regulation in Young Adults and Cognitively Diverse Older Adults

**DOI:** 10.1093/geroni/igaf122.051

**Published:** 2025-12-31

**Authors:** Claire Growney, Tammy English

**Affiliations:** Stanford University, Stanford, California, United States; Washington University in St. Louis, St. Louis, Missouri, United States

## Abstract

Effective emotion regulation (ER) often requires cognitive resources, but some strategies are theorized to be more cognitively demanding than others. Individuals can maintain emotional well-being by selecting ER strategies consistent with their available resources. Selection of low-demand strategies may be especially helpful for older adults experiencing accelerated cognitive decline. In this study, young adults (age 21–34, n = 66), cognitively normal older adults (CN; age 70–83, n = 90), and older adults with mild cognitive impairment (MCI; 70–84, n = 60) reported their perceived ER demands in a laboratory task and regarding their general use of ten ER strategies. We examined group differences in perceived demands as well as links between demands and frequency of use. Group differences varied by context: in the laboratory task, older adults with MCI reported higher demands than young and CN older adults, but reports of general demand perceptions revealed young adults perceive strategies as more demanding than older adults. Strategies varied significantly in their perceived cognitive demands, with strategies focused on enhancing positive emotion (e.g., savoring) rated as less demanding than those focused on reducing negative emotion (e.g., distraction). Multilevel models revealed that regulators used strategies less that they perceived as higher in cognitive demands among young adults (b=–0.20, SE = 0.05, p<.001) and CN older adults (b=–0.26, SE = 0.05, p<.001), but not older adults with MCI (b=–0.07, SE = 0.06, p=.220). Findings suggest that older adults may often have more ER expertise, but MCI may create ER difficulties, interfering with the ability to select easier to implement strategies.

